# RETRACTION: Effect of Mesenchymal Stromal Cells‐Derived Extracellular Vesicles as a Treatment to Heal Diabetic Wounds: A Meta‐Analysis

**DOI:** 10.1111/iwj.70222

**Published:** 2025-02-11

## Abstract

**RETRACTION:** ChangX. and LiJ. , “Effect of Mesenchymal Stromal Cells‐Derived Extracellular Vesicles as a Treatment to Heal Diabetic Wounds: A Meta‐Analysis,” International Wound Journal 20, no. 7 (2023): 2820–2829, 10.1111/iwj.14161.37015903 PMC10410336

The above article, published online on 04 April 2023, in Wiley Online Library (http://onlinelibrary.wiley.com/), has been retracted by agreement between the journal Editor in Chief, Professor Keith Harding; and John Wiley & Sons Ltd. Following an investigation by the publisher, all parties have concluded that this article was accepted solely on the basis of a compromised peer review process. In addition, the investigation found unattributed textual overlap between this article and another article by different authors [1]. The editors have therefore decided to retract the article. The authors did not respond to our notice regarding the retraction.

**Reference:**

[1] BaileyA. J. M. , LiH. , KirkhamA. M. , TieuA. , MagantiH. B. , ShorrR. , FergussonD. A. , LaluM. M. , ElomazzenH. , AllanD. S. , “MSC‐Derived Extracellular Vesicles to Heal Diabetic Wounds: a Systematic Review and Meta‐Analysis of Preclinical Animal Studies,” Stem Cell Reviews and Reports 18 (2022): 968–979, 10.1007/s12015-021-10164-4.33893619 PMC8064883

**RETRACTION:** X. Chang and J. Li , “Effect of Mesenchymal Stromal Cells‐Derived Extracellular Vesicles as a Treatment to Heal Diabetic Wounds: A Meta‐Analysis,” International Wound Journal 20, no. 7 (2023): 2820–2829, 10.1111/iwj.14161.37015903 PMC10410336

The above article, published online on 04 April 2023, in Wiley Online Library (http://onlinelibrary.wiley.com/), has been retracted by agreement between the journal Editor in Chief, Professor Keith Harding; and John Wiley & Sons Ltd. Following an investigation by the publisher, all parties have concluded that this article was accepted solely on the basis of a compromised peer review process. In addition, the investigation found unattributed textual overlap between this article and another article by different authors [1]. The editors have therefore decided to retract the article. The authors did not respond to our notice regarding the retraction.

## Reference

[1] A. J. M. Bailey , H. Li , A. M. Kirkham , A. Tieu , H. B. Maganti , R. Shorr , D. A. Fergusson , M. M. Lalu , H. Elomazzen , D. S. Allan , “MSC‐Derived Extracellular Vesicles to Heal Diabetic Wounds: a Systematic Review and Meta‐Analysis of Preclinical Animal Studies,” Stem Cell Reviews and Reports 18 (2022): 968–979, 10.1007/s12015-021-10164-4.33893619 PMC8064883

